# Month-of-Birth Effect on Muscle Mass and Strength in Community-Dwelling Older Women: The French EPIDOS Cohort

**DOI:** 10.3390/nu14224874

**Published:** 2022-11-18

**Authors:** Guillaume T. Duval, Anne-Marie Schott, Dolores Sánchez-Rodríguez, François R. Herrmann, Cédric Annweiler

**Affiliations:** 1 School of Medicine, Health Faculty, University of Angers, F-49100 Angers, France; 2 Department of Geriatric Medicine and Memory Clinic, Research Center on Autonomy and Longevity, University Hospital, F-49000 Angers, France; 3 UNIV ANGERS, UPRES EA 4638, F-49045 Angers, France; 4 Department IMER (Information Médicale, Evaluation, Recherche), Lyon University Hospital, EA 4129, Institut National de la Santé et de la Recherche Médicale, University of Lyon, U831 Lyon, France; 5 Geriatrics Department, Brugmann University Hospital, Université Libre de Bruxelles, B-1020 Brussels, Belgium; 6 Geriatrics Department, Rehabilitation Research Group, Institut Hospital del Mar d’Investigations Médiques (IMIM), E-08003 Barcelona, Spain; 7 Division of Public Health, Epidemiology and Health Economics, World Health Organization Collaborating Centre for Public Health Aspects of Musculo-Skeletal Health and Ageing, University of Liège, B-4000 Liège, Belgium; 8 Department of Rehabilitation and Geriatrics, Geneva University Hospitals, University of Geneva, CH-1226 Thônex, Switzerland; 9 Gérontopôle Autonomie Longévité des Pays de la Loire, F-44200 Nantes, France; 10 Department of Medical Biophysics, Robarts Research Institute, Schulich School of Medicine and Dentistry, The University of Western Ontario, London, ON N6A 5K8, Canada

**Keywords:** vitamin D, month of birth, muscle, pregnancy, older adults

## Abstract

**Background**. Vitamin D is involved in muscle health and function. This relationship may start from the earliest stages of life during pregnancy when fetal vitamin D relies on maternal vitamin D stores and sun exposure. Our objective was to determine whether there was an effect of the month of birth (MoB) on muscle mass and strength in older adults. **Methods**. Data from 7598 community-dwelling women aged ≥ 70 years from the French multicentric EPIDOS cohort were used in this analysis. The quadricipital strength was defined as the mean value of 3 consecutive tests of the maximal isometric voluntary contraction strength of the dominant lower limb. The muscle mass was defined as the total appendicular skeletal muscle mass measured using dual energy X-ray absorptiometry scanner. The MoB was used as a periodic function in regressions models adjusted for potential confounders including age, year of birth, latitude of recruitment center, season of testing, body mass index, number of comorbidities, IADL score, regular physical activity, sun exposure at midday, dietary protein intake, dietary vitamin D intake, use vitamin D supplements, history and current use of corticosteroids. **Results**. A total of 7133 older women had a measure of muscle strength (mean age, 80.5 ± 3.8 years; mean strength, 162.3 ± 52.1 N). Data on total ASM were available from 1321 women recruited in Toulouse, France (mean, 14.86 ± 2.04 kg). Both the sine and cosine functions of MoB were associated with the mean quadricipital strength (respectively β = −2.1, *p* = 0.045 and β = −0.5, *p* = 0.025). The sine function of MoB was associated with total ASM (β = −0.2, *p* = 0.013), but not the cosine function (β = 0.1, *p* = 0.092). Both the highest value of average quadricipital strength (mean, 163.4 ± 20.2 N) and the highest value of total ASM (15.24 ± 1.27 kg) were found among participants born in August. **Conclusions**. Summer-early fall months of birth were associated with higher muscle mass and strength in community-dwelling older women.

## 1. Introduction

Besides its classical role in the regulation of bone metabolism, vitamin D has many non-skeletal biological targets mediated by the vitamin D receptor (VDR), which is a specific vitamin D hormone receptor. Vitamin D is involved in the health and function of skeletal muscles, and serum vitamin D concentrations are positively associated with muscle mass and strength in adults [1]. The clinical relevance is that vitamin D deficiency leads to poorer muscular performance and physical deterioration [2]. Importantly, it was proposed that this relationship could start from the earliest stages of life, during pregnancy [3].

During gestation, the fetus is completely reliant on maternal vitamin D stores [4]. The maternal serum 25-hydroxyvitamin D (25OHD) concentration is highly correlated to that of the umbilical cord of the fetus [4]. Moreover, since 90% of maternal vitamin D is synthesized in the skin under the action of solar ultraviolet-B (UV-B) rays, the mother’s vitamin D status is mostly influenced by the season; with higher concentrations reported during summer and early fall [5]. Thus, in the absence of supplementation, fetal vitamin D concentration largely depends on the season of pregnancy [5]. This may explain why several conditions related to hypovitaminosis D have been previously linked to the month of birth (MoB); the children born in winter being more at risk of lower height and weight, and at risk of multiple sclerosis for instance [6].

Several studies have also brought evidence that the maternal serum 25OHD concentration and UV-B rays exposure during pregnancy influence the body composition in offspring, including bone mass, degree of adiposity, and muscle mass [7,8]. However, to the best of our knowledge, the relationship between the MoB and the muscle mass and function during adulthood has not been examined yet. We hypothesized that there could be an effect of the MoB on muscles in older adults, specifically that the summer-early fall MoB would be associated with better muscle mass and function. The objective of the present study was to determine whether there was an effect of the MoB on muscle mass and strength in community-dwelling older women.

## 2. Materials and Methods

### 2.1. Participants

We used for the present analysis data from the older women included in the ‘EPIDOS’ study (EPIDémiologie de l’OStéoporose), a French national prospective multicentric and observational cohort study originally designed to determine the risk factors for hip fracture among community-dwelling older women. Sampling and data collection procedures have been described in detail elsewhere [9]. In summary, from 1992 to 1994, 7598 women aged 70 years and older were recruited from electoral lists in five French cities (Amiens, Lyon, Montpellier, Paris and Toulouse). All included study participants had a full medical examination, which consisted of structured questionnaires, demographical measures including the month and year of birth, and a clinical examination.

### 2.2. Muscle Strength Measure

The maximal isometric voluntary contraction (MVC) strength of the dominant lower limb was measured with a strain gauge fixed to a chair, while the participant was seated, leg and ankle flexed at 90° angle. The leg tested was attached to the lever arm of the strain gauge and the seat height was adjusted to the leg length of the participant. Before carrying out the test, participants were offered to practice the isometric movement in order to warm up. Verbal instructions regarding the test procedure were given by a trained evaluator. Participants pushed as hard as possible against the dynamometer. Three MVC were recorded in Newton (N), and verbal encouragement was given each time to obtain the maximal score. The average MVC strength value was calculated from a set of three consecutive contractions and used for the present analysis.

### 2.3. Muscle Mass Measure

The total appendicular skeletal muscle mass (ASM) was measured using a dual energy X-ray absorptiometry (DXA) scanner (QDR 4500 W Hologic, Waltham, MA, USA) at enrollment only in the center of Toulouse, France. DXA measurements were performed by a trained technician, and the DXA machine was regularly calibrated. The total ASM was defined as the sum of the two upper and lower limb muscle masses, expressed in kilograms (kg). Data on the validity of body composition parameters of the EPIDOS-Toulouse cohort have previously been published [10].

### 2.4. Covariates

The following covariates were included as potential confounders in the statistical models: age, body mass index (BMI), number of comorbidities, regular physical activity, instrumental activities of daily living (IADL) score (from 0 to 8, best) [11], dietary protein and vitamin D intakes, use of vitamin D supplements, history of corticosteroids use, current use of corticosteroids, sun exposure at midday, season of evaluation, and study centers.

A physical examination and a health status questionnaire were conducted to assess comorbidities (i.e., hypertension, diabetes, dyslipidemia, coronary heart disease, chronic obstructive pulmonary disease, peripheral vascular disease, cancer, stroke, Parkinson’s disease and depression). Weight was measured with a beam balance scale, and height with a height gauge. BMI was calculated according to the formula: weight (kg)/height^2^ (m^2^). The practice of a physical activity was considered regular if the participants practiced at least one recreational physical activity (i.e., walking, gymnastics, cycling, swimming or gardening) for at least one hour per week for at least the past month. Medications taken regularly had to be brought by the participants to the clinical center during the assessment [9]. The dietary intakes of vitamin D and protein were estimated from a self-administered food frequency questionnaire, as previously published [12]. The cutaneous synthesis of vitamin D was estimated using the following standardized question: “When weather is nice, do you stay more than 15 min exposed to the sun (face and hands uncovered) between 11 a.m. and 3 p.m.?” (yes/no), as previously published [13]. Finally, the season of evaluation was recorded as follows: spring from 21 March to 20 June, summer from 21 June to 20 September, fall from 21 September to 20 December, winter from 21 December to 20 March.

### 2.5. Statistical Analysis

We provide here a post-hoc analysis of the EPIDOS cohort study. Firstly, the participants’ characteristics were summarized using means ± standard deviations or frequencies and percentages, as appropriate. As the number of observations was higher than 40, no transform was applied. Secondly, to determine the association of the MoB (independent variable) with muscle strength and muscle mass (dependent variables), we applied multiple linear regressions. The MoB was added as a periodic function: β1 × sin(2 π × MoB/12) + β2 × cos(2π × MoB/12), where π = 3.1415… and βi are regression coefficients. *p*-values < 0.05 were considered significant. All statistics were performed using Stata (version 14.1; College Station, TX, USA).

### 2.6. Ethics

Women participating in the study were included after having given their written informed consent for research. The study was conducted in accordance with the ethical standards set forth in the Helsinki Declaration (1983). The project was approved by the local ethics committee of each city.

## 3. Results

Among the 7598 women recruited in the EPIDOS cohort, 7133 had a measure of quadricipital strength (mean ± standard deviation age, 80.5 ± 3.8 years), and 1321 women (mean age, 80.3 ± 3.9 years; all from Toulouse, France) had a measure of total ASM. The mean quadricipital strength on the cohort was 162.3 ± 52.1 N, and the mean ASM 14.86 ± 2.04 kg (Table 1).

As indicated in Table 2, every MoB were represented, with a maximum of participants born in March (9.7%) and a minimum born in September (7.4%), with no significant difference.

**Figure 1:**
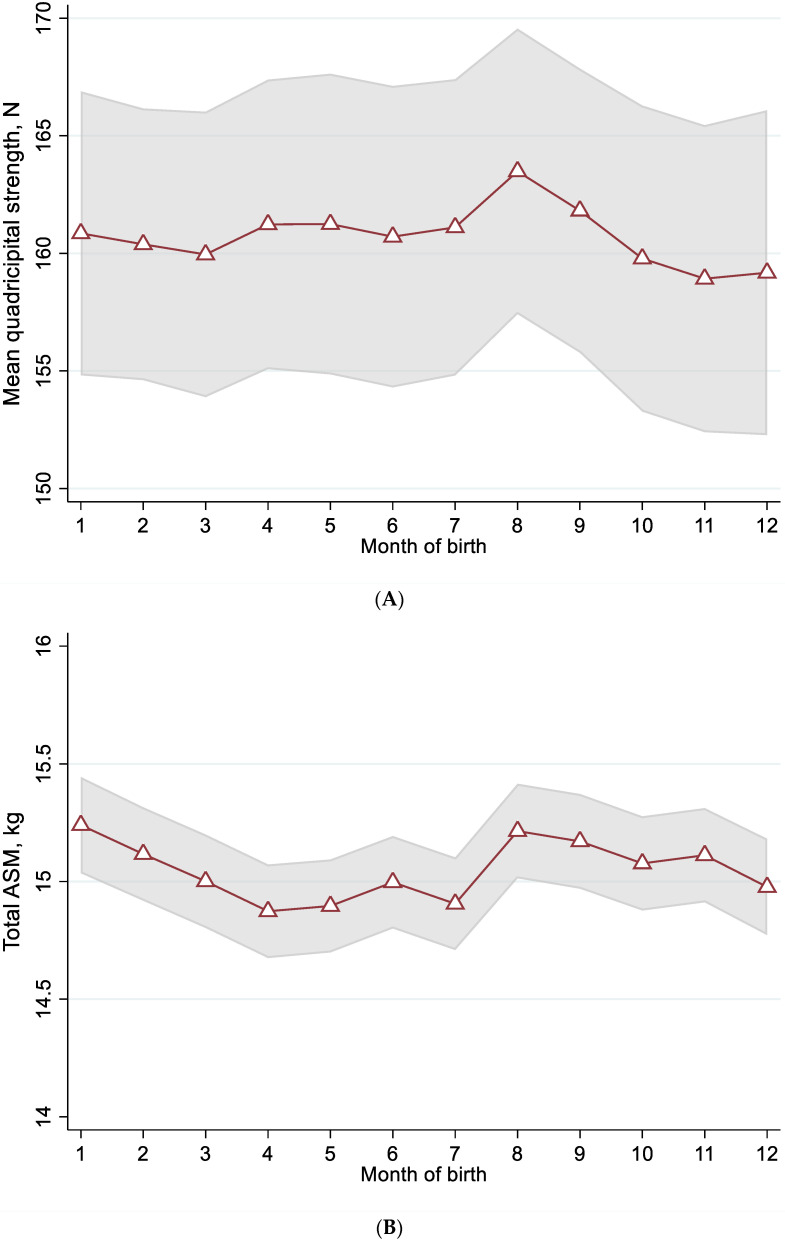
Trigonometric modelling by month of birth of (**A**) mean quadriceps strength (*n* = 7178), and (**B**) total appendicular skeletal muscle mass (ASM) (*n* = 1321). (**A**,**B**): Open triangles represent the average predicted values for respectively “Mean quadricipital strength” and “Total ASM” according to “Month of birth”. The shaded area represents the average predicted values plus or minus the median of the standard error of the prediction. The two models are adjusted for age, year of birth, latitude of recruitment centre, season of testing, body mass index, number of comorbidities, IADL score, regular physical activity, sun exposure at midday, dietary protein intake, dietary vitamin D intake, use of vitamin D supplements, history of corticosteroids use, and current use of corticosteroids.

Multiple linear regression models showed that both the sine and cosine functions of MoB were associated with the mean quadricipital strength (respectively fully adjusted β = −2.1 with *p* = 0.045, and fully adjusted β = −0.5 with *p* = 0.025), which means that the association between the MoB and mean quadricipital strength could be modeled using a combination of an inverted sine function and an inverted cosine function (Table 3, Figure 1A). Moreover, only the sine function of MoB was associated with the total ASM (fully adjusted β = −0.2, *p* = 0.013), but not the cosine function (fully adjusted β = 0.1, *p* = 0.092), which means that the association between the MoB and the total ASM was modeled by an inverted sine function (Table 3, Figure 1B). Both the highest predicted value of average quadricipital strength (mean, 163.4 ± 20.2 N) and the highest value of total ASM (15.24 ± 1.27 kg) were found among participants born in August (Table 3).

We also found that the BMI (β = 2.6, *p* = 0.001), a regular physical activity (β = 10.5, *p* = 0.002), and the IADL score (β = 5.9, *p* < 0.001) were positively associated with the mean quadricipital strength, although the number of comorbidities (β = −3.4, *p* = 0.019), the indication for vitamin D supplements (β = −5.2, *p* = 0.001) and the history of corticosteroids use (β = −13.0, *p* = 0.025) were inversely associated with the mean quadricipital strength. Finally, a more recent year of birth (β = 0.4, *p* = 0.001), the BMI (β = 0.3, *p* < 0.001), and the dietary protein intake (β = 0.1, *p* < 0.001) were positively associated with the total ASM, although the current use of corticosteroids (β = −0.8, *p* = 0.008), the need for vitamin D supplements (β = −0.5, *p* = 0.001) and the winter season of evaluation (β = −0.4, *p* = 0.016) were inversely associated with the total ASM.

## 4. Discussion

The main finding of this population-based study is that the month of birth was associated as a periodic function with the muscle mass and strength in a large cohort of community-dwelling older women, independently of all studied potential confounders. Summer-early fall MoBs, notably August, were associated with higher muscle mass and strength. These birthdates correspond to the participants born from pregnant women who were at the end of the second trimester and at the third trimester of pregnancy during the sunny period from May to August, i.e., the key moment for fetal muscle development.

Seasonality depends on individuals’ responses to seasonal fluctuations of environmental constraints. A widely studied seasonality is the effect of MoB on human traits. For instance, previous studies reported that the MoB influences a number of organic diseases [14], mood [15], as well as some morphological traits [16]. Birthweight, a marker of prenatal supplies, depends on the MoB [17]. This effect may be sustainable as birthweight is associated with muscle mass and strength throughout the life course from childhood to older age [18]; consistent with a potential influence of early life on long-term muscle development. Precisely, an effect of MoB on height and weight was also reported during childhood, and even in later life [16]. Weber et al. [19], using a large sample of conscripts in Austria, showed that males born between February to July were taller than those born in the remaining months. Similar observations were made by Banegas et al. [20], who found that Spanish male adults born in June/July were taller than those born in December/January. However, to our knowledge, we provide here the first evidence of a MoB effect on the muscle mass and function in older adults.

Several hypotheses may explain our finding. A first explanation is that the apparent MoB effect on muscles is explained by the influence of MoB on comorbidities. On the one hand, winter MoBs are associated with greater risks of heart disease, cerebrovascular disease, malignant neoplasms, and chronic respiratory diseases, with potential adverse consequences on physical activity and muscle mass and strength in adulthood [21,22]. On the other hand, the MoB may play a role in mood disorders due to the changing length of the photoperiod, and may influence the preference of individuals to plan activities rather in the morning or in the evening. According to Caci et al., people born in March/April are eveningness with a depressive mood tendency, although those born in September/October would be morningness and with impulsivity-related personality [23]. This may have an impact on daily physical activity and thus on muscle quality. However, the number of comorbidities, the IADL score, the practice of a regular physical activity and the sun exposure habits were used as potential confounders in the present analysis, and did not alter the association of MoB with muscle mass and function. Moreover, this first set of explanation could not account for the MoB-related morphological differences reported in offspring. Thus the alternative possibility of a direct MoB effect on muscles should be considered, based on the seasonal changes of maternal exposure to UV-B radiation during pregnancy and the subsequent changes of maternal serum vitamin D concentration. In French latitudes, the sun exposure of pregnant women is deemed insufficient between October and May to allow normal vitamin D concentration [24]. As the fetus is completely reliant on maternal vitamin D stores [4], and as 90% of maternal vitamin D is synthesized under the action of solar UV-B rays, the fetus experiences seasonal changes of 25OHD concentrations throughout gestation, with potential consequences on musculature.

Emerging evidence has shown that intrauterine exposure to 25OHD during pregnancy exerts a range of effects on development of skeletal muscle [1,25], probably through the modulation of the expression of muscle transcription factors [26]. Vitamin D results in the induction of myogenesis, proliferation, differentiation and cellular apoptosis [1], and may also participate in the control of protein synthesis in muscle cells by increasing the anabolic effect of insulin and leucine on muscle cells [1]. Recently, two studies in pigs found that improving maternal vitamin D status not only increased the number of fibers in longissimus dorsi of fetuses at day 90 of gestation [3], but also induced increased weight and muscle fiber cross-sectional area in psoas major and longissimus dorsi of weaning piglets [26]. In humans, maternal serum 25OHD concentration in pregnancy was positively associated with offspring height-adjusted handgrip strength and with offspring percent lean mass [27]. Evidence is also accruing that maternal serum 25OHD concentrations during pregnancy might influence offspring body composition in childhood [7,8]. Maternal antenatal serum 25OHD concentrations have been associated positively with bone mass [8] and negatively with fat mass [7]. Consistently, a population-based mother–offspring cohort study reported that maternal vitamin D status during late pregnancy could influence muscle strength of offspring at age 4 years [27]. Findings from the Mysore Parthenon Study, a prospective mother-offspring birth cohort in India, demonstrated greater arm muscle area at 5 and 9.5 years in children born to vitamin D–replete (serum 25OHD > 50 nmol/L) compared with vitamin D–depleted (25OHD < 50 nmol/L) mothers [7]. A positive association between maternal estimated UV-B exposure in the third trimester and offspring lean mass determined by DXA at 9.9 years of age was also observed in the Avon Longitudinal Study of Parents and Children (ALSPAC) [8]. All these observations support that vitamin D promotes both prenatal and postnatal skeletal muscle development, which may account for our findings, notably that people born in summer-early fall, when vitamin D status is optimal, are more prone to exhibit higher (i.e., better) muscle mass and strength in later life.

The implications for practice and research are manifold. First, our results support the idea that muscles changes related to early life hypovitaminosis D are persistent in adult age, suggesting a trait-like association between vitamin D status and muscles; consistent with the fact that hypertrophy of skeletal muscle fibers developed prenatally is the primary mechanism by which skeletal muscle growth occurs postnatally [28]. Second, they support the fact that the vitamin D status of pregnant women is crucial for fetal development and should be closely monitored in clinical routine. Offspring exposure to high levels of vitamin D appears essential for the muscles at the end of the second trimester and during the third trimester of pregnancy. Since 70% of pregnant women have hypovitaminosis D [4], these observations are relevant to public health and call for a precautionary approach based on maternal vitamin D monitoring and eventual repletion. Third, they provide a strong rationale for conducting clinical trials in pregnant women, which is expected to reveal long-term effects of vitamin D supplements on body composition, behavioral development and physical function. We propose that future clinical trials should focus on pregnancies that give birth in winter-early spring. In this perspective, our findings participate in further elucidating the profile of ideal target populations, which is an important step to provide effective guidelines on the proper use of vitamin D supplements during pregnancy.

Our results confirmed that the use of corticosteroids is associated with reduced muscle mass and strength [29], although more frequent physical activity is associated with increased strength [30], and higher dietary intakes of proteins are associated with increased muscle mass [31]. These consensual results strengthen the consistency of our study and confirm the relevance of the MoB effect we found on muscle mass and strength.

The strengths of this study include a large sample of older adults recruited in five centers with various latitudes. Additionally, the participants recruited were all born in a large period, from 1893 to 1922, in an era without vitamin supplementation D policy for pregnant women. This could have emphasized the role of seasonality and sun exposure. We also had the opportunity to measure the muscle mass with DXA, which is more accurate and relevant than anthropometric measures such as the calf circumference. Finally, regression models were applied to measure adjusted associations. Regardless, some potential limitations of our study should be considered. Firstly, the MoB is a proxy measure of maternal vitamin D status during pregnancy, and no information was available on the actual vitamin D status of the participants’ mothers during pregnancy. Secondly, the study cohort was restricted to relatively vigorous older women who may be unrepresentative of older adults in general, especially regarding the musculature. The study participants may have been also more motivated, with a greater interest in personal health issues, than the general population of older adults. Thirdly, the use of an observational design precludes inferring any causal inference. Fourthly, although we were able to control for important characteristics that could modify the association between MoB and muscles, residual potential confounders, such as the latitude of the birthplaces, might still be present. Then, the ASM was measured only in one center, Toulouse, and one latitude, 43°36′ N. Finally, no information on eventual premature birth was available, although premature delivery appears to be more frequent in mothers deficient in vitamin D [32].

## 5. Conclusions

In conclusion, our results show for the first time to our knowledge that the month of birth is associated as a periodic function with the muscle mass and strength in a large cohort of community-dwelling older women, with potential consequences on various health outcomes including diabetes mellitus, falls, fractures, and all-cause mortality [33]. The summer-early fall months of birth were associated with higher muscle mass and strength in late life. This suggests that enhancing maternal vitamin D status during pregnancy whether through sun exposure, diet or supplementation, might improve prenatal and postnatal muscle development. This new orientation may offer a powerful mechanism to better understand the muscular changes in older adults, and to act on their healthcare early in life by setting up vitamin D supplementation in pregnant women. However, formal testing of this hypothesis in an interventional setting should be undertaken before the development of any formal clinical recommendations.

## Figures and Tables

**Table 1:** Summary of the participants’ characteristics (*n* = 7133).

Characteristics	Cohort	
	Summary Value	(95% CI)
**Demographical measures**		
Age, years	80.5 ± 3.8 (70–98)	(80.4; 80.6)
Year of birth	1912 ± 4 (1893–1922)	-
Recruitment center, n (%)		
Amiens (49°54′ N)	1488 (20.9)	(20.0; 21.8)
Paris (48°51′ N)	1457 (20.4)	(19.5; 21.3)
Lyon (45°45′ N)	1365 (19.1)	(18.2; 20.0)
Montpellier (43°36′ N)	1492 (20.9)	(20.0; 21.8)
Toulouse (43°36′ N)	1331 (18.7)	(17.8; 19.6)
Season of testing, n (%)		
Spring	1922 (26.9)	(25.9; 27.9)
Summer	1876 (26.3)	(25.3; 27.3)
Fall	1843 (25.8)	(24.8; 26.8)
Winter	1492 (20.9)	(20.0; 21.8)
**Clinical measures**		
Body mass index, kg/m^2^	25.3 ± 4.0 (14.5–42.1)	(25.2; 25.4)
Number of comorbidities, n (%)	3.4 ± 2.0 (0–24)	(3.36; 3.44)
IADL score, /8	6.3 ± 1.3 (0–8)	(6.27; 6.33)
Regular physical activity, n (%)	3490 (48.9)	(47.7; 50.1)
Sun exposure at midday, n (%)	3609 (50.6)	(49.4; 51.8)
Dietary protein intake, g/day	69.6 ± 16.2 (8.7–162.3)	(69.2; 70.0)
Dietary vitamin D intake, µg/week	63.1 ± 31.3 (0–278.0)	(62.3; 63.9)
Use of vitamin D supplements, n (%)	985 (13.8)	(13.0; 14.6)
History of corticosteroids use, n (%)	455 (6.4)	(5.8; 7.0)
Current use of corticosteroids, n (%)	146 (2.0)	(1.7; 2.3)
**Muscles measures**		
Mean quadricipital strength, N	162.3 ± 52.1 (17.3–507.3)	(161.1; 163.5)
Total ASM, kg *	14.86 ± 2.04 (9.33–22.78)	(14.75; 14.97)

Data presented as mean ± standard deviation [range] where applicable. ASM: appendicular skeletal muscle mass; CI: confidence interval; SD: standard deviation; IADL: Instrumental Activities of Daily Living; *: data available for 1321 participants.

**Table 2:** Months of birth among the participants (*n* = 7133).

Month of Birth	Cohort			
	n	Percentage	(95% CI)	Cumulative Percentage
January	630	8.83	(8.17; 9.49)	8.83
February	602	8.43	(7.79; 9.07)	17.27
March	690	9.67	(7.79; 9.07)	26.95
April	568	7.96	(7.33; 8.59)	34.91
May	603	8.45	(7.80; 9.10)	43.36
June	573	8.03	(7.40; 8.66)	51.39
July	625	8.76	(8.10; 9.42)	60.16
August	598	8.38	(7.74; 9.02)	68.54
September	524	7.35	(6.74; 7.96)	75.89
October	527	7.39	(6.78; 8.00)	83.27
November	585	8.20	(7.56; 8.84)	91.48
December	608	8.52	(7.87; 9.17)	100.00

**Table 3:** Average values of muscles’ measures predicted with the trigonometric modelling by month of birth.

Month of Birth	Mean Quadricipital Strength, N	Total ASM, kg
January	161.0 ± 20.8	15.06 ± 1.20
February	160.0 ± 19.5	15.13 ± 1.19
March	160.0 ± 19.6	15.01 ± 1.17
April	161.0 ± 19.8	14.89 ± 1.22
May	161.2 ± 19.6	14.91 ± 1.15
June	160.9 ± 19.6	15.01 ± 1.18
July	160.6 ± 20.5	14.93 ± 1.24
August	163.4 ± 20.2	15.24 ± 1.27
September	161.6 ± 20.6	15.20 ± 1.29
October	159.8 ± 20.5	15.10 ± 1.19
November	158.8 ± 19.8	15.13 ± 1.21
December	159.0 ± 19.2	14.99 ± 1.21
Beta for sin (*p*-value)	β = −2.1, *p* = 0.045	β = −0.2, *p* = 0.013
Beta for cos (*p*-value)	β = −0.5, *p* = 0.025	β = 0.1, *p* = 0.092

Data presented as mean ± standard deviation.

## Data Availability

Patient level data are freely available from the corresponding author at cedric.annweiler@chu-angers.fr. There is no personal identification risk within this anonymized raw data, which is available after notification and authorization of the competent authorities.

## Abbreviations

25OHD	25-hydroxyvitamin D
ASM	Appendicular skeletal muscle mass
BMI	body mass index
kg	kilograms
DXA	Dual Energy X-ray Absorptiometry
IADL	instrumental activities of daily living
MoB	month of birth
N	Newton
SD	standard deviations
UV-B	ultraviolet-B rays
VDR	vitamin D receptor
